# A cut finite element method for elliptic bulk problems with embedded surfaces

**DOI:** 10.1007/s13137-019-0120-z

**Published:** 2019-01-29

**Authors:** Erik Burman, Peter Hansbo, Mats G. Larson, David Samvin

**Affiliations:** 1 Mathematics, University College London, London, UK; 2 Mechanical Engineering, Jönköping University, Jönköping, Sweden; 3 Mathematics and Mathematical Statistics, Umeå University, Umeå, Sweden

**Keywords:** Finite element, Unfitted, Embedded, Fractures, 65M60, 74S05, 76S05

## Abstract

We propose an unfitted finite element method for flow in fractured porous media. The coupling across the fracture uses a Nitsche type mortaring, allowing for an accurate representation of the jump in the normal component of the gradient of the discrete solution across the fracture. The flow field in the fracture is modelled simultaneously, using the average of traces of the bulk variables on the fractures. In particular the Laplace–Beltrami operator for the transport in the fracture is included using the average of the projection on the tangential plane of the fracture of the trace of the bulk gradient. Optimal order error estimates are proven under suitable regularity assumptions on the domain geometry. The extension to the case of bifurcating fractures is discussed. Finally the theory is illustrated by a series of numerical examples.

## Introduction

We consider a model Darcy creeping flow problem with low permeability in the bulk and with embedded interfaces with high permeability. Our approach is based on the Nitsche extended finite element of Hansbo and Hansbo (2002), which however did not include transport on the interface. Here, we follow Capatina et al. (2016) and let a suitable mean of the solution on the interface be affected by a transport equation see also Burman et al. (2015). We present a complete a priori analysis and consider the important extension to bifurcating fractures.

The flow model we use is essentially the one proposed in Capatina et al. (2016). More sophisticated models have been proposed, e.g., in Angot et al. (2009), Formaggia et al. (2014), Frih et al. (2008) and Martin et al. (2005), in particular allowing for jumps in the solution across the interfaces. To allow for such jumps, one can either align the mesh with the interfaces, as in, e.g., Berrone et al. (2017) and Hægland et al. (2009), or use extended finite element techniques, cf. Burman et al. (2015), Capatina et al. (2016), D’Angelo and Scotti (2012) and Del Pra et al. (2017).

In previous work Burman et al. (2017) we used a continuous approximation with the interface equations simply added to the bulk equation, which does not allow for jumps in the solution. This paper presents a more complex, but more accurate, discrete solution to the problem. To reduce the technical detail of the arguments we consider a semi-discretization of the problem where we assume that the integrals on the interface and the subdomains separated by the interface can be evaluated exactly. The results herein can be extended to the fully discrete setting, with a piecewise affine approximation of the fracture using the analysis detailed in Burman et al. (2016).

An outline of the paper is as follows: in Sect. 2 we formulate the model problem, its weak form, and investigate the regularity properties of the solution, in Sect. 3 we formulate the finite element method, in Sect. 4 we derive error estimates, in Sect. 5 we extend the approach to the case of bifurcating fractures, and in Sect. 6 we present numerical examples including a study of the convergence and a more applied example with a network of fractures.

## The model problem

**Fig. 1:**
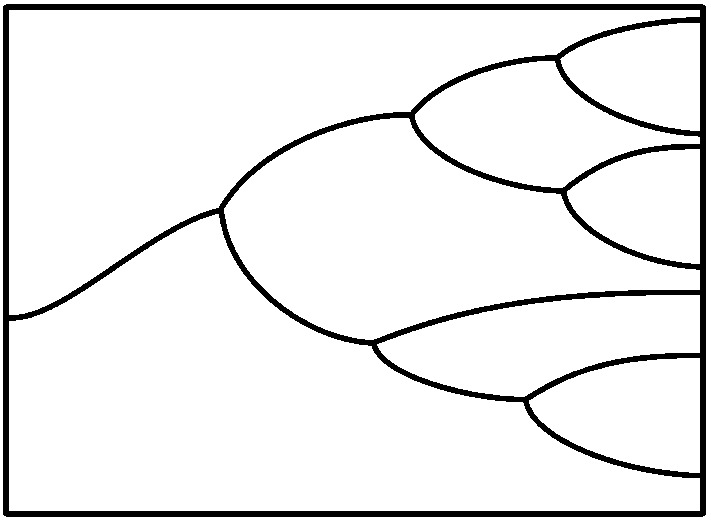
Schematic figure of bifurcating fractures

In this section we introduce our model problem. First we present the strong form of the equations and then we derive the weak form that is used for the finite element modelling. We discuss the regularity properties of the solution and show that if the fracture is sufficiently smooth the problem solution, restricted to the subdomains partitioning the global domain, has a regularity that allows for optimal approximation estimates for piecewise affine finite element methods (Fig. 1).

### Strong and weak formulations

Let $\varOmega$ be a convex polygonal domain in ${\mathbb {R}}^d$, with $d=2$ or 3. Let $\varGamma$ be a smooth embedded interface in $\varOmega$, which partitions $\varOmega$ into two subdomains $\varOmega _1$ and $\varOmega _2$. We consider the problem: find the pressure $u:\varOmega \rightarrow {\mathbb {R}}$ such that

2.1 $$\begin{aligned} -\nabla \cdot a \nabla u&= f&\qquad&\text {in }\varOmega _i, i=1,2 \end{aligned}$$

2.2 $$\begin{aligned} -\nabla _\varGamma \cdot a_\varGamma {\nabla _\varGamma u}&= f_\varGamma - \llbracket n \cdot a \nabla u \rrbracket&\qquad&\text {on }\varGamma \end{aligned}$$

2.3 $$\begin{aligned}{}[u]&= 0&\qquad&\text {on } \varGamma \end{aligned}$$

2.4 $$\begin{aligned} u&=0&\qquad&\text {on }\partial \varOmega \end{aligned}$$

Here

2.5 $$\begin{aligned}{}[v] = v_1 - v_2, \qquad \llbracket n \cdot a \nabla v \rrbracket = n_1 \cdot a_1 \nabla v_1 + n_2 \cdot a_2 \nabla v_2 \end{aligned}$$

where $v_i = v |_{H^1(\varOmega _i)}$, $n_i$ is the exterior unit normal to $\varOmega _i$, $a_i$ are positive bounded permeability coefficients, for simplicity taken as constant, and $0\le a_\varGamma <\infty$ is a constant permeability coefficient on the interface. Note that it follows from (2.3) that *v* is continuous across $\varGamma$ while from (2.2) we conclude that the normal flux is in general not continuous across $\varGamma$. Note also that taking $a_\varGamma = 0$ and $f_\varGamma =0$ corresponds to a standard Poisson problem with possible jump in permeability coefficient across $\varGamma$.

To derive the weak formulation of the system we introduce the $L^2$-scalar product over a domain $X \subset {\mathbb {R}}^d$, or $X \subset {\mathbb {R}}^{d-1}$. For $u,v \in L^2(X)$ let

2.6 $$\begin{aligned} (u,v)_X = \int _X u\,v~\hbox {d}X \end{aligned}$$

with the associated norm $\Vert u\Vert _X = (u,u)_X^{1/2}$. Multiplying (2.1) by

2.7 $$\begin{aligned} v \in V :=\left\{ v\in H^1(\varOmega ) ;\quad v\vert _\varGamma \in H^1(\varGamma )\right\} , \end{aligned}$$

integrating by parts over $\varOmega _i$, and using (2.2), we obtain

2.8 $$\begin{aligned} (f,v)_\varOmega&= -(\nabla \cdot a \nabla u, v)_{\varOmega _1} -(\nabla \cdot a \nabla u, v)_{\varOmega _2} \end{aligned}$$

2.9 $$\begin{aligned}&=(a \nabla u,\nabla v)_{\varOmega _1} + (a \nabla u,\nabla v)_{\varOmega _2} - (\llbracket n \cdot a \nabla u \rrbracket , v )_\varGamma \end{aligned}$$

2.10 $$\begin{aligned}&= (a \nabla u,\nabla v)_\varOmega - (f_\varGamma + \nabla _\varGamma \cdot a_\varGamma \nabla _\varGamma u, v )_\varGamma \end{aligned}$$

2.11 $$\begin{aligned}&= (a \nabla u,\nabla v)_\varOmega + ( a_\varGamma \nabla _\varGamma u, \nabla _\varGamma v )_\varGamma - (f_\varGamma ,v)_\varGamma \end{aligned}$$

We thus arrive at the weak formulation: find $u \in V$ such that

2.12 $$\begin{aligned} (a \nabla u,\nabla v)_\varOmega + ( a_\varGamma \nabla _\varGamma u, \nabla _\varGamma v )_\varGamma = (f,v)_\varOmega + (f_\varGamma ,v)_\varGamma \qquad \forall v \in V \end{aligned}$$

Observing that *V* is a Hilbert space with scalar product

2.13 $$\begin{aligned} a(v,w) = (a \nabla v,\nabla w)_\varOmega + ( a_\varGamma \nabla _\varGamma v, \nabla _\varGamma w)_\varGamma \end{aligned}$$

and associated norm $\Vert v \Vert ^2_a = a(v,v)$ it follows from the Lax–Milgram lemma that there is a unique solution to (2.12) in *V* for $f\in H^{-1}(\varOmega )$ and $f_\varGamma \in H^{-1}(\varGamma )$.

### Regularity properties

To prove optimality of our finite element method we need that the exact solution is sufficiently regular. However since the normal fluxes jump over the interface the solution can not have square integrable weak second derivatives. If the interface is smooth however we will prove that the solution restricted to the different subdomains $\varOmega _1$, $\varOmega _2$ and $\varGamma$ is regular. The upshot of the unfitted finite element method is that this local regularity is sufficient for optimal order approximation. More precisely we have the elliptic regularity estimate 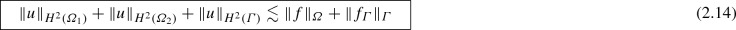


#### Proof

Let $$u_i \in H^1_0(\varOmega _i)$$ solve

2.15 $$\begin{aligned} (a_i \nabla u_i, \nabla v)_{\varOmega _i} = (f,v)_{\varOmega _i}\qquad \forall v \in H^1_0(\varOmega _i) \end{aligned}$$

Then we have

2.16 $$\begin{aligned} \Vert u_i \Vert _{H^2(\varOmega _i)} \lesssim \Vert f \Vert _{\varOmega _i} \qquad i = 1,2 \end{aligned}$$

Let $u = u_\varGamma + u_1 + u_2$ where $u_\varGamma$ satisfies

2.17 $$\begin{aligned} -\nabla _\varGamma \cdot a_\varGamma \nabla _\varGamma u _\varGamma&= f_\varGamma + \llbracket n \cdot a \nabla (u_\varGamma + u_1 + u_2)\rrbracket \end{aligned}$$

2.18 $$\begin{aligned}&= f_\varGamma + \llbracket n \cdot a \nabla u_\varGamma \rrbracket + n_1 \cdot a \nabla u_1 + n_2 \cdot a \nabla u_2 \qquad \text {on } \varGamma \end{aligned}$$

and

2.19 $$\begin{aligned} -\nabla \cdot a \nabla u_\varGamma = 0 \qquad \text {on }\varOmega _i, i=1,2 \end{aligned}$$

Using (2.16) we conclude that

2.20 $$\begin{aligned} n_i \cdot a \nabla u_i |_\varGamma \in H^{1/2}(\varGamma )\qquad i=1,2 \end{aligned}$$

Furthermore, using that $u_\varGamma \in H^1(\varGamma )$, which follows from the fact that $u_\varGamma \in V$ it follows that $u_\varGamma |_{\varOmega _i} \in H^{3/2}(\varOmega _i)$, $i=1,2,$ and thus

2.21 $$\begin{aligned} \llbracket n \cdot a \nabla u_\varGamma \rrbracket \in H^{1/2}(\varGamma ) \end{aligned}$$

Since the right hand side of (2.18) is in $L^2(\varGamma )$ we may use elliptic regularity for the Laplace Beltrami operator to confirm that

2.22 $$\begin{aligned} u_\varGamma |_\varGamma \in H^2(\varGamma ) \end{aligned}$$

Collecting the bounds we obtain the refined regularity estimate

2.23 $$\begin{aligned} \Vert u_\varGamma \Vert _{H^2(\varGamma )} + \sum _{i=1}^2 \left( \Vert u_\varGamma \Vert _{H^{5/2}(\varOmega _i)} + \Vert u_i \Vert _{H^2(\varOmega _i)} \right) \lesssim \Vert f \Vert _\varOmega + \Vert f_\varGamma \Vert _\varGamma \end{aligned}$$

where we note that we have stronger control of $u_\varGamma$ on the subdomains. $\square$

## The finite element method

### The mesh and finite element space

Let ${\mathcal {T}}_h$ be a quasi-uniform conformal mesh, consisting of shape regular elements with mesh parameter $h\in (0,h_0]$, on $\varOmega$ and let

3.1 $$\begin{aligned} {\mathcal {T}}_{h,i} = \left\{ T \in {\mathcal {T}}_h : T \cap \varOmega _i \ne \emptyset \right\} \quad i=1,2 \end{aligned}$$

be the active meshes associated with $\varOmega _i$, $i=1,2.$ Let $V_h$ be a finite element space consisting of continuous piecewise polynomials on ${\mathcal {T}}_h$ and define

3.2 $$\begin{aligned} V_{h,i} = V_h |_{{\mathcal {T}}_{h_i}} \qquad i =1,2 \end{aligned}$$

and

3.3 $$\begin{aligned} W_h = V_{h,1} \oplus V_{h,2} \end{aligned}$$

To $v =v_1 \oplus v_2 \in W_h$ we associate the function ${\widetilde{v}} \in L^2(\varOmega )$ such that ${\widetilde{v}}|_{\varOmega _i} = v_i|_{\varOmega _i}$, $i=1,2$. In general, we simplify the notation and write ${\widetilde{v}} = v$. Finally, we use ${\mathcal {T}}_h(\varGamma )$ to denote the set of elements intersected by $\varGamma$.

### Derivation of the method

To derive the finite element method we follow the same approach as when introducing the weak formulation, but taking care to handle the boundary integrals that appear due to the discontinuities in the approximation space.

Testing the exact problem with $v \in W_h$ and integrating by parts over $\varOmega _1$ and $\varOmega _2$ we find that

3.4 $$\begin{aligned}&\displaystyle (f,v)_{\varOmega _1} + (f,v)_{\varOmega _2} \end{aligned}$$

3.5 $$\begin{aligned}&\displaystyle = (-\nabla \cdot a \nabla u,v)_{\varOmega _1} + (-\nabla \cdot a \nabla u,v)_{\varOmega _2} \end{aligned}$$

3.6 $$\begin{aligned}&\displaystyle = (a\nabla u,\nabla v)_\varOmega - (\langle n\cdot a \nabla u \rangle , [v] )_\varGamma - (\llbracket n\cdot a \nabla u\rrbracket ,\langle v \rangle _* )_\varGamma \end{aligned}$$

3.7 $$\begin{aligned}&\displaystyle = (a\nabla u,\nabla v)_\varOmega - (\langle n\cdot a \nabla u \rangle , [v] )_\varGamma - (\nabla _\varGamma \cdot a_\varGamma \nabla _\varGamma u,\langle v \rangle _*)_\varGamma - (f_\varGamma ,\langle v \rangle _* )_\varGamma \qquad \end{aligned}$$

3.8 $$\begin{aligned}&\displaystyle = (a \nabla u,\nabla v)_\varOmega - (\langle n\cdot a \nabla u \rangle , [v] )_\varGamma + (a_\varGamma \nabla _\varGamma u,\nabla _\varGamma \langle v \rangle _* )_\varGamma - (f_\varGamma ,\langle v \rangle _* )_\varGamma \qquad \end{aligned}$$

3.9 $$\begin{aligned}&\displaystyle = (a \nabla u,\nabla v)_\varOmega - (\langle n\cdot a \nabla u \rangle , [v] )_\varGamma - ([u],\langle n\cdot a \nabla v \rangle )_\varGamma \end{aligned}$$

3.10 $$\begin{aligned}&\quad \displaystyle + (a_\varGamma \nabla _\varGamma u,\nabla _\varGamma \langle v \rangle _* )_\varGamma - (f_\varGamma ,\langle v \rangle _* )_\varGamma \end{aligned}$$

where in the last identity we symmetrized using the fact that $[u]=0$. We also used the identity

3.11 $$\begin{aligned}{}[v w ] = [v]\langle w \rangle + \langle v \rangle _* [w] \end{aligned}$$

where the averages are defined by

3.12 $$\begin{aligned} \langle w \rangle = \kappa _1 w_1 + \kappa _2 w_2, \qquad \langle w \rangle _* = \kappa _2 w_1 + \kappa _1 w_2 \end{aligned}$$

with $$\kappa _1+\kappa _2=1$$ and $$0\le \kappa _i \le 1$$.

Introducing the bilinear forms

3.13 $$\begin{aligned}&\displaystyle a_{\varOmega }(v,w)= (a \nabla v,\nabla w)_{\varOmega _1}{+}(a \nabla v,\nabla w)_{\varOmega _2} {-} (\langle n\cdot a \nabla v \rangle , [w] )_\varGamma - ([v],\langle n\cdot a \nabla w \rangle )_\varGamma \qquad \end{aligned}$$

3.14 $$\begin{aligned}&\displaystyle a_{h,\varGamma }(v,w) = (a_\varGamma \nabla _\varGamma \langle v \rangle _*,\nabla _\varGamma \langle w \rangle _* )_\varGamma , \end{aligned}$$

3.15 $$\begin{aligned}&\displaystyle l_h(v) = (f,v)_\varOmega + (f_\varGamma ,\langle v \rangle _*)_\varGamma \end{aligned}$$

the above formal derivation leads to the following consistent formulation for discontinuous test functions *w*. For $u\in W = H^1(\varOmega )\cap H^{3/2}(\varOmega _1) \cap H^{3/2}(\varOmega _2) \cap H^1(\varGamma )$ the solution to (2.12) there holds 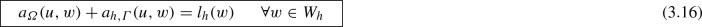


Observe that we have modified $a_{h,\varGamma }$ by introducing the average $\langle v \rangle _*$ also in the left factor. This changes nothing when applied to a smooth solution, but will allow also to apply the form to the discontinuous discrete approximation space. The subscript *h* in the form indicates that it is modified to be well defined for the discontinuous approximation space. The definition of *W* is motivated by the fact that the trace terms should be well defined, for instance,

3.17 $$\begin{aligned} \displaystyle (\langle n \cdot a \nabla v \rangle , [w] )_\varGamma\lesssim & {} \left( \sum _{i=1}^2 \Vert v_i \Vert ^2_{H^1(\partial \varOmega _i)} \right) ^{1/2} \left( \sum _{i=1}^2 \Vert w_i \Vert ^2_{\partial \varOmega _i} \right) ^{1/2} \end{aligned}$$

3.18 $$\begin{aligned} \displaystyle\lesssim & {} \left( \sum _{i=1}^2 \Vert v_i \Vert ^2_{H^{3/2}( \varOmega _i)} \right) ^{1/2} \left( \sum _{i=1}^2 \Vert w_i \Vert ^2_{H^1(\varOmega _i)} \right) ^{1/2} \end{aligned}$$

where we used the trace inequalities $\Vert v \Vert _{H^s(\partial \varOmega _i)} \lesssim \Vert v \Vert _{H^{s+1/2}(\varOmega _i)}$ for $s>0$ and $\Vert w \Vert _{\partial \varOmega _i} \lesssim \Vert w \Vert _{H^{1/2 + \epsilon }(\varOmega _i)} \lesssim \Vert w \Vert _{H^1(\varOmega _i)}$ for $\epsilon >0$.

### The finite element method

The finite element method that we propose is based on the formulation (3.16). However, using this formulation as it stands does not lead to a robust approximation method. Indeed we need to ensure stability of the formulation through the addition of consistent penalty terms. First we need to enforce continuity of the discrete solution across $\varGamma$. To this end we introduce an augmented version of $a_\varOmega$,

$$\begin{aligned} a_h(v,w)= a_\varOmega (v,w) + \beta h^{-1} ([v],[w])_\varGamma \end{aligned}$$

with $\beta$ a positive parameter. Since the exact solution $u \in H^1(\varOmega )$, there holds $a_\varOmega (u,w) = a_h(u,w)$. Secondly, to obtain stability independently of how the interface cuts the computational mesh and for strongly varying permeabilities $a_1$, $a_2$ and $a_\varGamma$ we also need some penalty terms in a neighbourhood of the interface. We define

$$\begin{aligned} s_h(v,w) = s_{h,1}(v_1,w_1)+s_{h,2}(v_2,w_2) \end{aligned}$$

where

3.19 $$\begin{aligned} s_{h,i}(v_i,w_i)=\gamma h([n \cdot a \nabla v_i],[n \cdot a \nabla w_i])_{{\mathcal {F}}_{h,i}}\qquad i=1,2 \end{aligned}$$

where $\gamma$ is a positive parameters and ${\mathcal {F}}_{h,i}$ is the set of interior faces in ${\mathcal {T}}_{h,i}$ that belongs to an element $T\in {\mathcal {T}}_{h,i}$ which intersects $\varGamma$, see Fig. 6. Observe that for $u \in H^2(\varOmega _1\cup \varOmega _2)$, $s_h(u,v)=0$ for all $v \in W_h$.

Collecting the above bilinear forms in

3.20 $$\begin{aligned} A_h(v,w) = a_h(v,w) + s_h(v,w) + a_{h,\varGamma }(v,w) \end{aligned}$$

the finite element method reads: 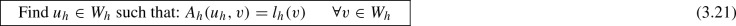


## Analysis of the method

In this section we derive the basic error estimates that the solution of the formulation (3.21) satisfies. The technical detail is kept to a minimum to improve readability. In particular, we assume that the bilinear forms can be computed exactly and that $\varGamma$ fulfils the conditions of Hansbo and Hansbo (2002). For a more complete exposition in a similar context we refer to Burman et al. (2016).

### Properties of the bilinear form

For the analysis it is convenient to define the following energy norm

4.1 $$\begin{aligned} |||v |||_h^2 = \sum _{i=1}^2\left( \Vert a_i^{1/2} \nabla v \Vert _{\varOmega _i}^2 + | v|^2_{s_i}\right) + c_a h \Vert \langle n \cdot a \nabla v \rangle \Vert ^2_{\varGamma } + \beta h^{-1} \Vert [v ] \Vert ^2_{\varGamma } + \Vert a_\varGamma \nabla _\varGamma \langle v \rangle _* \Vert ^2_{\varGamma }\nonumber \\ \end{aligned}$$

where $| v|_{s_i}=s_i(v,v)^{1/2}$ and $c_a$ is a constant fulfilling $c_a \sim a_{\min }^{-1}$, cf. Lemma 4.1 below.

#### Lemma 4.1

The form $A_h$, defined in (3.20), satisfies the following bounds:

- $$A_h$$ is continuous 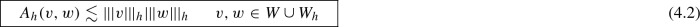 where *W* was introduced in (3.16).
- $A_h$ is coercive on $W_h$, 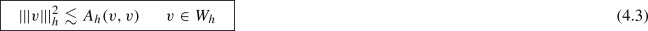 provided $\beta$ is large enough.

#### Proof

The first estimate (4.2) follows directly from the Cauchy–Schwarz inequality. To show (4.3) we recall the following inequalities:

4.4 $$\begin{aligned} \Vert a_i^{1/2} \nabla v \Vert ^2_{{\mathcal {T}}_{h,i}}\lesssim & {} \Vert a_i^{1/2}\nabla v \Vert ^2_{\varOmega _i} + | v |^2_{s_{h,i}} \quad { (\hbox {see Burman and Hansbo 2012})} \qquad \end{aligned}$$

4.5 $$\begin{aligned} h \Vert \langle n \cdot a \nabla v \rangle \Vert ^2_\varGamma\lesssim & {} \sum _{i=1}^2 \Vert \kappa _i a_i \nabla v \Vert ^2_{{\mathcal {T}}_{h,i}(\varGamma )} { \quad (\hbox {see Hansbo and Hansbo 2002})} \end{aligned}$$

In (4.5) we used the notation ${\mathcal {T}}_{h,i}(\varGamma ):= \{T \in {\mathcal {T}}_{h,i}: T \cap \varGamma \ne \emptyset \}$. To prove the claim observe that for all $v \in W_h$

4.6 $$\begin{aligned} A_h(v,v)= & {} \sum _{i=1}^2\left( \Vert a_i^{1/2} \nabla v \Vert _{\varOmega _i}^2 + | v|^2_{s_i}\right) + \beta h^{-1} \Vert [v ] \Vert ^2_{\varGamma }\nonumber \\&+\, \Vert a_\varGamma \nabla _\varGamma \langle v \rangle _* \Vert ^2_{\varGamma }-2\left( \langle n\cdot a \nabla v \rangle , [v]\right) _\varGamma \end{aligned}$$

Using (4.4) and (4.5) we obtain the following bound on the fluxes

4.7 $$\begin{aligned} h \Vert \langle n \cdot a \nabla v \rangle \Vert ^2_{\varGamma } \le C \sum _{i=1}^2 \kappa _i a_i \left( \Vert a_i^{1/2}\nabla v \Vert ^2_{\varOmega _i} + | v |^2_{s_{h,i}}\right) \end{aligned}$$

Now assume that $\kappa _i a_i \le a_{\min }:=\min _{i\in \{1,2\}} a_i$, for instance one may take $\kappa _1 = a_2/(a_1+a_2)$ and $\kappa _2=a_1/(a_1+a_2)$ then

4.8 $$\begin{aligned} 2\left( \langle n\cdot a \nabla v \rangle , [v]\right) _\varGamma&\le 2 a_{\min }^{-1/2} h^{1/2} \Vert \langle n \cdot a \nabla v \rangle \Vert _{\varGamma } a_{\min }^{1/2} h^{-1/2} \Vert [v]\Vert _\varGamma \end{aligned}$$

4.9 $$\begin{aligned}&\le \varepsilon h a_{\min }^{-1} \Vert \langle n \cdot a \nabla v \rangle \Vert ^2_{\varGamma }+ a_{\min } h^{-1} \varepsilon ^{-1} \Vert [v]\Vert ^2_\varGamma \end{aligned}$$

4.10 $$\begin{aligned}&\le C \varepsilon \sum _{i=1}^2 \left( \Vert a_i^{1/2}\nabla v \Vert ^2_{\varOmega _i} + | v |^2_{s_{h,i}}\right) + a_{\min } h^{-1} \varepsilon ^{-1} \Vert [v]\Vert ^2_\varGamma \end{aligned}$$

It follows that

4.11 $$\begin{aligned} A_h(v,v)\ge & {} (1 - C \varepsilon ) \sum _{i=1}^2\left( \Vert a_i^{1/2} \nabla v \Vert _{\varOmega _i}^2 + | v|^2_{s_i}\right) \nonumber \\&\quad + \left( \beta - a_{\min }/\varepsilon \right) h^{-1} \Vert [v ] \Vert ^2_{\varGamma } + \Vert a^{1/2}_\varGamma \nabla _\varGamma \langle v \rangle _* \Vert ^2_{\varGamma } \end{aligned}$$

The bound (4.3) now follows taking $\varepsilon = 1/(2C)$ and $\beta > 2 C a_{\min }$ and by applying once again (4.7), taking $c_a \sim a_{\min }^{-1}$. $\square$

A consequence of the bound (4.3) is the existence of a unique solution to (3.21).

#### Lemma 4.2

The linear system defined by the formulation (3.21) is invertible.

#### Proof

Follows from Lax–Milgram’s lemma. $\square$

### Interpolation

For $\delta >0$ let $E_i:H^s(\varOmega _i) \rightarrow H^s(\varOmega )$ be a continuous extension operator $s>0$. We define the interpolation operator

4.12 $$\begin{aligned} \pi _h:L^2(\varOmega ) \ni v \mapsto \pi _{h,1} v_1 \oplus \pi _{h,2} v_2 \in V_{h,1} \oplus V_{h,2} = W_h \end{aligned}$$

where $\pi _{h,i}: L^2(\varOmega _i) \, {\ni }\, v_i \mapsto \pi ^{SZ}_{h,i} {E_i} v_i \in V_{h,i}$, $i=1,2,$ and $\pi _h^{SZ}$ is the Scott–Zhang interpolation operator. We then have the interpolation error estimate 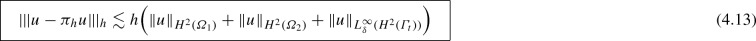 where, with $\rho _\varGamma$ the signed distance function associated with $\varGamma$,

4.14 $$\begin{aligned} \varGamma _t = \{x \in \varOmega : \rho _\varGamma (x) = t \}, \qquad |t|\le {\delta } \end{aligned}$$

and

4.15 $$\begin{aligned} \Vert v \Vert _{L^\infty _\delta \left( H^s(\varGamma _t)\right) } = \sup _{|t|\le \delta } \Vert v \Vert _{\left( H^s(\varGamma _t)\right) } \end{aligned}$$

#### Proof

To prove the estimate (4.13) we use a trace-inequality on functions in $H^1({\mathcal {T}}_h(\varGamma ))$ (i.e., with $\Vert \cdot \Vert _{{\mathcal {T}}_h(\varGamma )}$ the broken $H^1$-norm over the elements intersected by $\varGamma$),

4.16 $$\begin{aligned} \Vert v_i\Vert _\varGamma \lesssim h^{-1/2} \Vert E_i v_i\Vert _{{\mathcal {T}}_h(\varGamma )} + h^{1/2} \Vert \nabla E_i v_i\Vert _{{\mathcal {T}}_h(\varGamma )} \end{aligned}$$

see Hansbo and Hansbo (2002), then interpolation on ${\mathcal {T}}_h(\varGamma )$ and finally we use the stability of the extension operator $E_i$. First observe that by using the trace inequality (4.16) we obtain, with $v=u - \pi _h u$

4.17 $$\begin{aligned} \Vert (\beta h)^{-1/2} [v]\Vert _\varGamma + c_a h \Vert \langle n \cdot a \nabla v \rangle \Vert _{\varGamma }\lesssim & {} \sum _{i=1}^2\left( h^{-1} \Vert v_i \Vert _{{\mathcal {T}}_h(\varGamma )}+\Vert \nabla v_i \Vert _{{\mathcal {T}}_h(\varGamma )})\right. \nonumber \\&\left. +\,h \Vert \nabla ^2 v_i \Vert _{{\mathcal {T}}_h(\varGamma ))}\right) \end{aligned}$$

Using standard interpolation for the Scott–Zhang interpolation operator we get the bound

4.18 $$\begin{aligned} \Vert (\beta h)^{-1/2} [v]\Vert _\varGamma + c_a h \Vert \langle n \cdot a \nabla v \rangle \Vert _{\varGamma }\lesssim & {} h \sum _{i=1}^2 | E_i u_i|_{H^2( {\mathcal {T}}_h(\varGamma ))} \nonumber \\\lesssim & {} h \sum _{i=1}^2 | u_i|_{H^2(\varOmega _i)} \end{aligned}$$

where we used the stability of the extension operator in the last inequality. The bound

4.19 $$\begin{aligned} |u - \pi _h u|_{s_i} \lesssim h \sum _{i=1}^2 |a_i^{1/2} u_i|_{H^2(\varOmega _i)} \end{aligned}$$

follows similarly using element-wise trace inequalities follows by interpolation (c.f. Burman and Hansbo 2012). The interpolation error estimate for the terms due to the Laplace–Beltrami operator on $\varGamma$ is a bit more delicate. We use a trace inequality to conclude that

4.20 $$\begin{aligned} \Vert a^{1/2}_\varGamma \nabla _\varGamma \langle u - \pi _h u \rangle _\star \Vert _\varGamma ^2&\lesssim \sum _{i=1}^2 \Vert a^{1/2}_\varGamma \nabla _\varGamma ( u_i - \pi _{h,i} u_i ) \Vert _\varGamma ^2 \end{aligned}$$

4.21 $$\begin{aligned}&\lesssim \sum _{i=1}^2 h^{-1}\Vert \nabla ( u_i - \pi _{h,i} u_i) \Vert _{{\mathcal {T}}_h(\varGamma )}^2 + h \Vert \nabla ^2 ( u_i - \pi _{h,i} u_i) \Vert _{{\mathcal {T}}_h(\varGamma )}^2 \end{aligned}$$

4.22 $$\begin{aligned}&\lesssim \sum _{i=1}^2 h \Vert \nabla ^2 u_i \Vert _{ {\mathcal {T}}_h(\varGamma )}^2 \end{aligned}$$

4.23 $$\begin{aligned}&\lesssim \delta h \Vert u \Vert ^2_{L^\infty _\delta (H^2(\varGamma _t))} \end{aligned}$$

Observing that we may take $\delta \sim h$ the estimate follows. $\square$

Comparing (4.13) with (2.23) we see that we have a small mismatch between the regularity that we can prove and that required to achieve optimal convergence. In view of this we need to assume a slightly more regular solution for the $H^1$-error estimates below. The sub optimal regularity also interferes in the $L^2$-error estimates. Here we need to use (2.23) on the dual solution and in this case the additional regularity of the estimate (4.13) is not available. Instead we need to find the largest $\zeta \in [0,1]$ such that $|||u - \pi _h u |||_h \lesssim h^\zeta \sum _{i=1}^2 \Vert u \Vert _{H^2(\varOmega _i)}$, which will result in a suboptimality by a power of $1-\zeta$ in the convergence order in the $L^2$-norm. Revisiting the analysis above up to (4.22) we see that

4.24 $$\begin{aligned} |||u - \pi _h u |||_h&{} \lesssim h \sum _{i=1}^2 | u_i|_{H^2(\varOmega _i)} \nonumber \\&\quad +\sum _{i=1}^2 h^{-1/2}\Vert \nabla ( u_i - \pi _{h,i} u_i) \Vert _{{\mathcal {T}}_h(\varGamma )} + h^{1/2} \Vert \nabla ^2 ( u_i - \pi _{h,i} u_i) \Vert _{{\mathcal {T}}_h(\varGamma )} \end{aligned}$$

4.25 $$\begin{aligned}&{} \lesssim \left( h + h^{1/2}\right) \sum _{i=1}^2 | u_i|_{H^2(\varOmega _i)} \end{aligned}$$

### Error estimates

#### Theorem 4.1

If *u* is the solution to (2.1)–(2.4), satisfying $u\in H^2(\varOmega _1\cup \varOmega _2)\cup L^\infty _{{\delta }}(H^2(\varGamma _t))$, and $u_h$ is the finite element approximation defined by (3.21), then 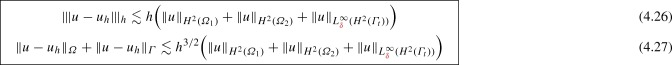


#### Proof

(4.26). Splitting the error and using the interpolation error estimate we have

4.28 $$\begin{aligned} |||u - u_h |||_h&\le |||u - \pi _h {u}|||_h + |||\pi _h u - u_h |||_h \end{aligned}$$

Using coercivity (4.3), Galerkin orthogonality and continuity (4.2) the second term can be estimated as follows

4.29 $$\begin{aligned} |||\pi _h u - u_h |||_h^2&\lesssim A_h( \pi _h u - u_h, \pi _h u - u_h ) \end{aligned}$$

4.30 $$\begin{aligned}&= A_h( \pi _h u - u, \pi _h u - u_h ) \end{aligned}$$

4.31 $$\begin{aligned}&\le |||\pi _h u - u |||_h |||\pi _h u - u_h |||_h \end{aligned}$$

and thus applying the approximation result (4.13) we conclude that

4.32 $$\begin{aligned} |||u - u_h |||_h&\lesssim |||u - \pi _h u |||_h \end{aligned}$$

4.33 $$\begin{aligned}&\lesssim h \Big ( \Vert u \Vert _{H^2(\varOmega _1)} + \Vert u \Vert _{H^2(\varOmega _2)} + \Vert u \Vert _{L^\infty _{{\delta }}(H^2(\varGamma _t))}\Big ) \end{aligned}$$

(4.27). Consider the dual problem

4.34 $$\begin{aligned} A(v,\phi ) = ( v, \psi )_\varOmega + (v,\psi _\varGamma ) \qquad \forall v \in V \end{aligned}$$

and recall that by (2.23) we have the elliptic regularity

4.35 $$\begin{aligned} \sum _{i=1}^2 \Vert \phi \Vert _{H^2(\varOmega _i)} + \Vert \phi _\varGamma \Vert _{H^2(\varGamma )} \lesssim \sum _{i=1}^2 \Vert \psi _i \Vert _{\varOmega _i} + \Vert \psi _\varGamma \Vert _{\varGamma } \end{aligned}$$

Setting $v= e = u - u_h$ and using Galerkin orthogonality, followed by the continuity (4.2) and the suboptimal approximation estimate (4.24) on $|||\phi - \pi _h \phi |||_h$ we get

4.36 $$\begin{aligned} (e,\psi )_\varOmega + (e,\psi _\varGamma )_\varGamma&=A_h(e,\phi ) \end{aligned}$$

4.37 $$\begin{aligned}&=A_h(e,\phi - \pi _h \phi ) \end{aligned}$$

4.38 $$\begin{aligned}&\le |||e |||_h |||\phi - \pi _h \phi |||_h \end{aligned}$$

4.39 $$\begin{aligned}&\lesssim |||e |||_h h^{1/2}\left( \sum _{i=1}^2 \Vert \phi \Vert _{H^2(\varOmega _i} + \Vert \phi _\varGamma \Vert _{H^2(\varGamma )} \right) \end{aligned}$$

4.40 $$\begin{aligned}&\lesssim h^{1/2} |||e |||_h \left( \sum _{i=1}^2 \Vert \psi _i \Vert _{\varOmega _i} + \Vert \psi _\varGamma \Vert _{\varGamma } \right) . \end{aligned}$$

In the last step we used the elliptic regularity estimate (4.35) for the dual problem. Setting $\psi _ i = e_i / \Vert e_i\Vert _{\varOmega _i}$ and $\psi _\varGamma = e_\varGamma /\Vert e_\varGamma \Vert _{\varGamma }$ estimate (4.27) follows. $\square$

#### Remark 4.1

As noted before the error estimate in the $L^2$-norm is suboptimal with a power 1 / 2. To improve on this estimate we would need to sharpen the regularities required for the approximation estimate (4.13). This appears to be highly non-trivial since the interpolation of *u* and $u_\varGamma$ can not be separated when both are interpolated using the bulk unknowns. Therefore we did not manage to exploit the stronger control that we have on the harmonic extension of $u_\varGamma$ in (2.23). Note however that if separate fields are used on the fracture and in the bulk domains we would recover optimal order convergence in $L^2$.

#### Remark 4.2

Using the stronger control of the regularity of the harmonic extension provided by (2.23) we may however establish an optimal order $L^2$ error estimate for the solution on $\varGamma$, 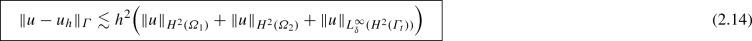


## Extension to bifurcating fractures

In the case most common in applications, fractures bifurcate, leading to networks of interfaces in the bulk. It is straightforward to include this case in the method above and we will discuss the method with bifurcating fractures below. The analysis can also be extended under suitable regularity assumptions, but becomes increasingly technical. We leave the analysis of the methods modelling flow in fractured media with bifurcating interfaces to future work.

### The model problem

**Fig. 2:**
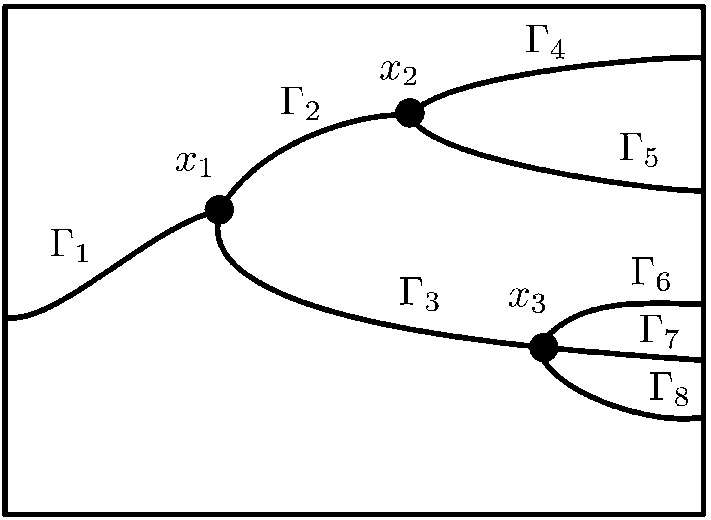
Notation for bifurcating fractures

*Description of the domain* Let us for simplicity consider a two dimensional problem with a one dimensional interface. We define the following:

- Let the interface $\varGamma$ be described as a planar graph with nodes ${\mathcal {N}} = \{ x_i \}_{i\in I_N}$ and edges ${\mathcal {E}} =\{\varGamma _j\}_{j\in I_E}$, where $I_N$, $I_E$ are finite index sets, and each $\varGamma _j$ is a smooth curve between two nodes with indexes $I_N(j)$. Note that edges only meet in nodes.
- For each $i \in I_N$ we let $I_E(i)$ be the set of indexes corresponding to edges for which $x_i$ is a node. For each $i \in I_N$ we let $I_E(i)$ be the set of indexes *j* such that $x_i$ is an end point of $\varGamma _j$, see Fig. 2.
- The graph $\varGamma$ defines a partition of $\varOmega$ into *N* subdomains $\varOmega _i$, $i=1,\ldots ,N$.

*The Kirchhoff condition* The governing equations are given by (2.1)–(2.4) together with two conditions at each of the nodes $x_i \in {\mathcal {N}}$, the continuity condition

5.1 $$\begin{aligned} u_{\varGamma _k} (x_i) = u_{\varGamma _l} (x_i) \qquad \forall k,l \in I_E(i) \end{aligned}$$

and the Kirchhoff condition

5.2 $$\begin{aligned} \sum _{j \in I_{E}(i)} (t_{\varGamma _j} \cdot a_{\varGamma _j} \nabla _{\varGamma _j} u_{\varGamma _j}) |_{x_j} =0 \end{aligned}$$

where $t_{\varGamma _j} (x_i)$ is the exterior tangent unit vector to $\varGamma _j$ at $x_i$. Note that in the special case when a node $x_i$ is an end point of only one curve the Kirchhoff condition becomes a homogeneous Neumann condition.

### The finite element method

*Forms associated with the bifurcating interface* Let $V_\varGamma = \{v \in C(\varGamma ) : v \in H^1(\varGamma _j), j \in I_E\}$ and $V = H^1_0(\varOmega ) \cap V_\varGamma$. We proceed as in the derivation (2.8)–(2.11) of the weak problem (2.12) in the standard case. However, when we use Green’s formula on $\varGamma$ we proceed segment by segment as follows

5.3 $$\begin{aligned}&\sum _{j \in I_E} -\left( \nabla _{\varGamma _j} \cdot a_{\varGamma _j} \nabla _{\varGamma _j} u_j,\langle v_j \rangle _*\right) _{\varGamma _j} \nonumber \\&\quad = \sum _{j \in I_E} \left( a_{\varGamma _j} \nabla _{\varGamma _j} u, \nabla _{\varGamma _j} \langle v \rangle _* \right) _{\varGamma _j} - \sum _{j \in I_E} \sum _{i \in I_N \left( j\right) } \left( t_i \cdot a_{\varGamma _j}\nabla _{\varGamma _j} u,\langle v \rangle _*\right) _{x_i} \end{aligned}$$

5.4 $$\begin{aligned}&\quad = \sum _{j \in I_E} \left( a_{\varGamma _j} \nabla _{\varGamma _j} u,\nabla _{\varGamma _j} \langle v\rangle _*\right) _{\varGamma _j} -\sum _{i \in I_N } \sum _{j \in I_E\left( i\right) } \left( t_i \cdot a_{\varGamma _j}\nabla _{\varGamma _j} u,\langle v \rangle _* - \langle \langle v\rangle _* \rangle _i\right) _{x_i} \end{aligned}$$

where we changed the order of summation and used the Kirchhoff condition (5.2) to subtract the nodal average

5.5 $$\begin{aligned} \langle v \rangle _i = \sum _{j\in I_E(i)} \kappa _j^{\varGamma } v_j(x_i) \end{aligned}$$

where $0<\kappa _i^{\varGamma }$, and $\sum _{j \in I_E(i)} \kappa _j^{\varGamma } = 1$. Note that when a node $x_i$ is an end point of only one curve the contribution from $x_i$ is zero, because in that case we have $\langle \langle v\rangle _* \rangle _i|_{x_i} - \langle v \rangle _* = 0$ since there is only one element in $I_E(i)$, and thus we get the standard weak enforcement of the homogeneous Neumann condition.

Symmetrizing and adding a penalty term we obtain the form

5.6 $$\begin{aligned} a_{h,\varGamma }\left( v,w\right)&= \sum _{j \in I_E} \left( a_{\varGamma _j} \nabla _{\varGamma _j} \langle v\rangle _*,\nabla _{\varGamma _j} \langle w\rangle _*\right) _{\varGamma _j} \nonumber \\&\quad -\sum _{i \in I_N } \sum _{j\in I_E\left( i\right) } \left( t_j \cdot a_{\varGamma _j}\nabla _{\varGamma _j} \langle v\rangle _* ,\langle w \rangle _* - \langle \langle v\rangle _* \rangle _i\right) _{x_i} \nonumber \\&\quad -\sum _{i \in I_N } \sum _{j \in I_E\left( i\right) } \left( \langle v \rangle _* - \langle \langle v\rangle _* \rangle _i, t_j \cdot a_{\varGamma _j}\nabla _{\varGamma _j} \langle w \rangle \right) _{x_i} \nonumber \\&\quad + \sum _{i \in I_N } \sum _{j\in I_E\left( i\right) } \beta ^{\varGamma } h^{-1} \left( \langle v \rangle _* - \langle \langle v\rangle _* \rangle _i , \langle w \rangle _* - \langle \langle w\rangle _* \rangle _i\right) _{x_i} \end{aligned}$$

where $\beta ^\varGamma$ is a stabilisation parameter with the same function as $\beta$. A similar derivation can be performed for a two dimensional bifurcating fracture embedded into ${\mathbb {R}}^3$, see Hansbo et al. (2017) for further details.

To ensure coercivity we add a stabilization term of the form

5.7 $$\begin{aligned} s_{h,\varGamma }(v,w) = \sum _{j \in I_E} s_{h,\varGamma _j}(v,w) \end{aligned}$$

where

5.8 $$\begin{aligned} s_{h,\varGamma _j}\left( v,w\right) = \left( [\nabla _{\varGamma _j} \langle v \rangle _*] , [ \nabla _{\varGamma _j} \langle w\rangle _*] \right) _{{\mathcal {X}}_h\left( \varGamma _j\right) } \end{aligned}$$

and ${\mathcal {X}}_h(\varGamma _j)$ is the set of points

5.9 $$\begin{aligned} \varGamma _j \cap {\mathcal {F}}_h(x_i) \end{aligned}$$

where ${\mathcal {F}}_h(x_i)$ is the set of interior faces in the patch of elements ${\mathcal {N}}_h(T(x_i))$ and $T(x_i)$ is an element such that $x_i \in T$.

We finally define the form $A_{h,\varGamma }$ associated with the bifurcating crack by

5.10 $$\begin{aligned} A_{h,\varGamma }(v,w) = a_{h,\varGamma }(v,w) + s_{h,\varGamma } (v,w ) \qquad \forall v \in W_h \end{aligned}$$

*The method* Define

5.11 $$\begin{aligned} W_h = \bigoplus _{i=1}^N V_{h,i} \end{aligned}$$

where $V_{h,i} = V_h|_{{\mathcal {T}}_{h,i}}$. The method takes the form: find $u_h \in W_h$ such that

5.12 $$\begin{aligned} A_h(u_h,v) = l_h(v)\quad \forall v \in W_h \end{aligned}$$

where

5.13 $$\begin{aligned} A_h(v,w) = \sum _{i=1}^N A_{h,i}(v,w) + A_{h,\varGamma }(v,w) \end{aligned}$$

and

5.14 $$\begin{aligned} A_{h,i} (v,w) = a_{h,i}(v,w) + s_{h,i}(v,w) \end{aligned}$$

## Numerical examples

### Implementation details

**Fig. 3:**
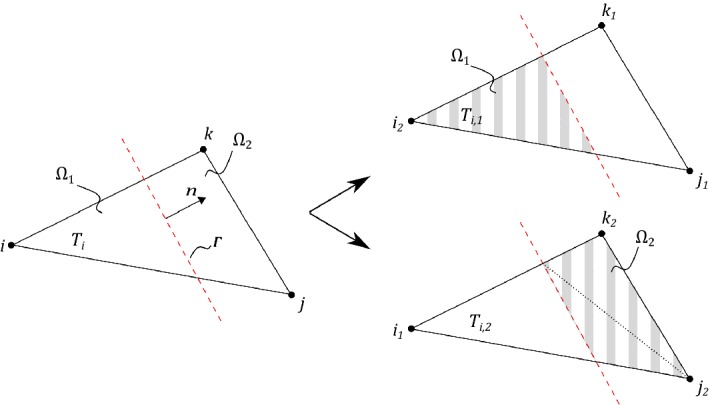
The split of a triangle without bifurcation point

**Fig. 4:**
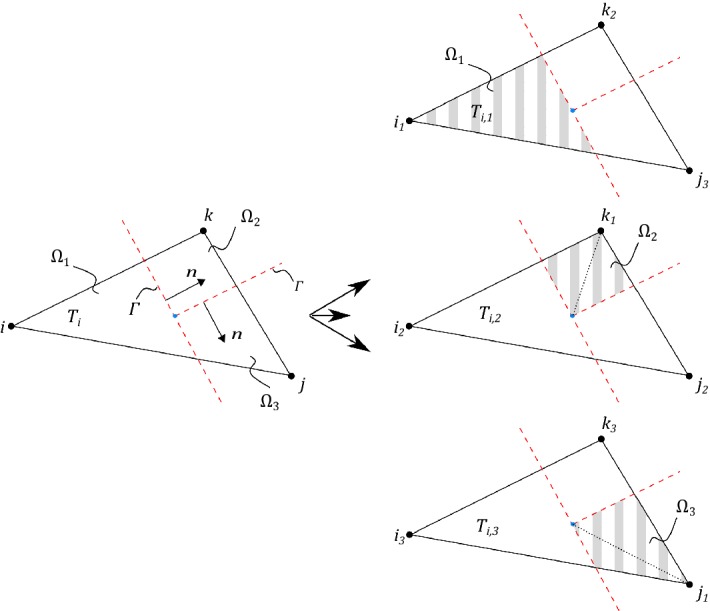
The split of a triangle with bifurcation point

We will employ piecewise linear triangles and extend the implementation approach proposed in Hansbo and Hansbo (2002) to include also bifurcating fractures. Recall that $${\mathcal {T}}_h(\varGamma ) $$ denotes the set of elements intersected by $$\varGamma $$, where each side of the intersection belongs to $$\varOmega _1$$ and $$\varOmega _2$$, respectively. For each element in $$ T_i \in {\mathcal {T}}_h(\varGamma ) $$, we assign elements $$T_{i,1} \in {\mathcal {T}}_{h,1}$$ and $$T_{i,2} \in {\mathcal {T}}_{h,2}$$ by overlapping the existing element $$T_i \in {\mathcal {T}}_h(\varGamma )$$ using the *same* nodes from the original triangulation. Elements $$T_{i,1} $$ and $$T_{i,2} $$ coincide geometrically, see Fig. 3. To ensure continuity, we used the same process on the neighboring elements and checked if new nodes had already been assigned. For each bifurcation point, two approaches can be adapted. Either by letting the bifurcation point coincide with a node or by the less straight-forward approach to overlap the existing element $$T_i \in {\mathcal {T}}_h(\varGamma ) $$ into $$T_{i,1} $$, $$T_{i,2} $$ and $$T_{i,3} $$, see Fig. 4. For simplicity of implementation, we have here chosen to let the bifurcating point coincide with a node. The triangles $$T_i \notin {\mathcal {T}}_h(\varGamma )$$ were handled in the usual way. The stabilization (3.19) was only applied to the *cut sides* of the elements which in all examples was sufficient for stability.

### Example 1: No flow in fracture

We consider an example on $\varOmega = (0,1) \times (0,1)$, from Hansbo and Hansbo (2002). We solved the example with an added bifurcation point. For the added fracture, we denote the diffusion coefficient by $a_{\varGamma _1}$. The exact solution is given by

**Fig. 5:**
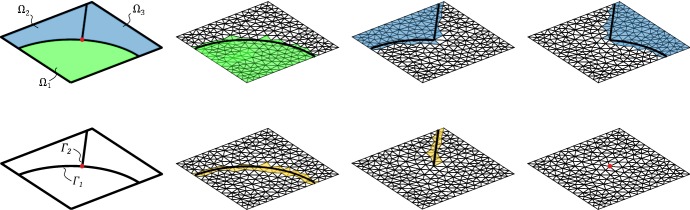
Active meshes with two embedded fractures, Example 1

**Fig. 6:**
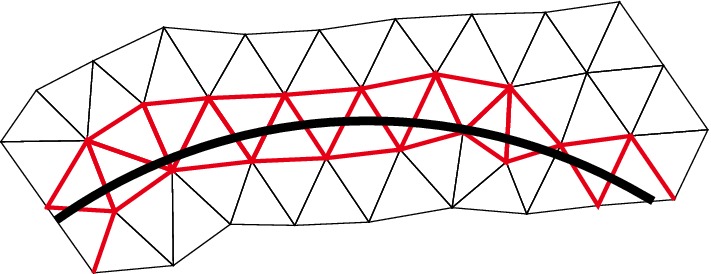
The red edges indicates the selection for computing stabilization terms asscording to (3.19) (color figure online)

**Fig. 7:**
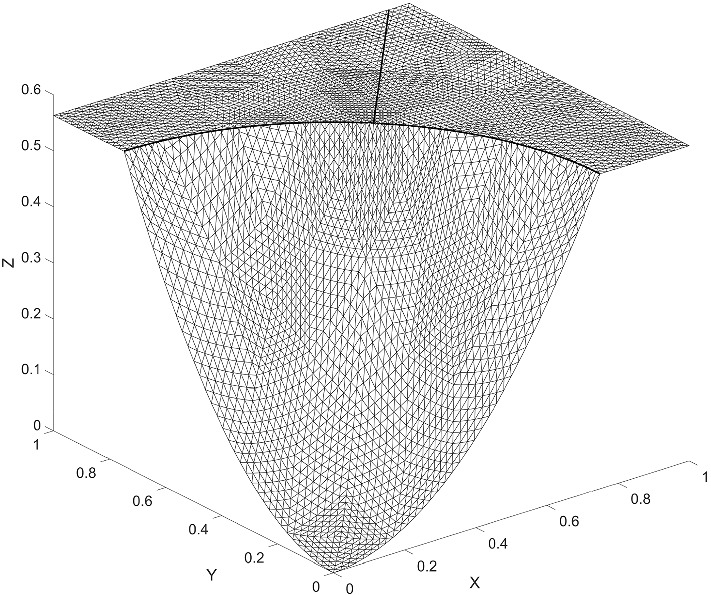
Elevation of the approximate solution with two embedded fractures, Example 1

**Fig. 8:**
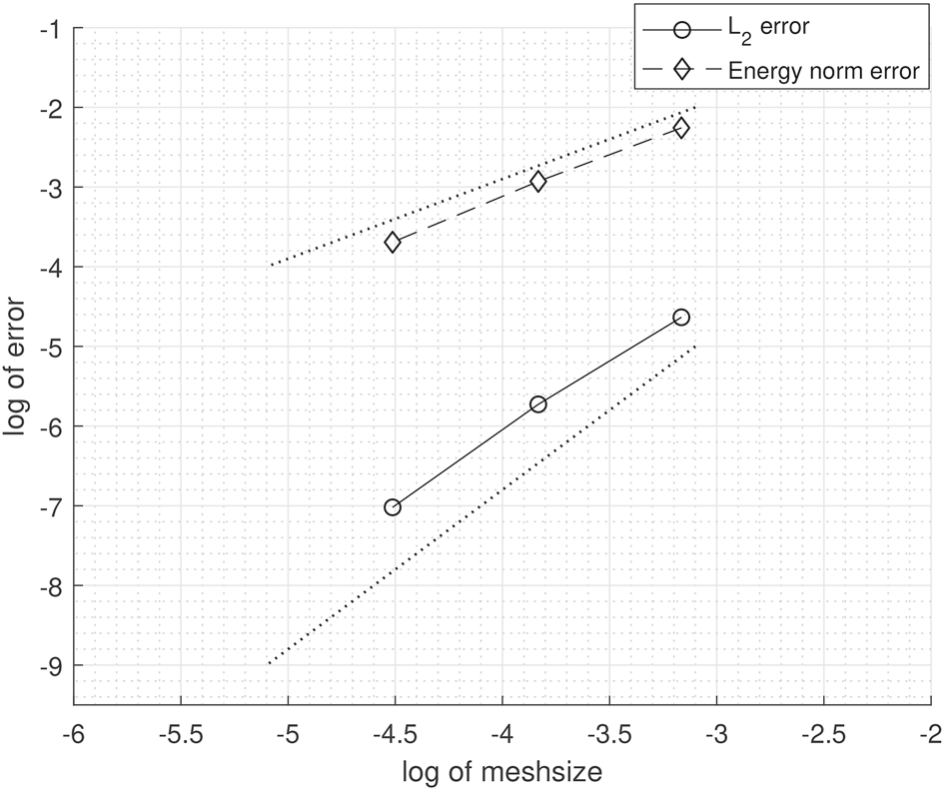
$$ L_2$$-norm and energy-norm convergence using natural logarithm with two embedded fractures, Example 1. Dotted lines signify optimal convergence. Inclination 1:1 for energy-norm and 2:1 for $$ L_2$$-norm

6.1 $$\begin{aligned} u(x, y) = {\left\{ \begin{array}{ll} \frac{r^2}{a_1}, &{} \quad \text {if}\quad r \leqslant r_0 \\ \frac{r^2}{a_2} - \frac{r_0^2}{a_2} + \frac{r_0^2}{a_1}, &{} \quad \text {if} \quad r > r_0 \end{array}\right. } \end{aligned}$$

where $$r = \sqrt{x^2 + y^2}$$. We chose $$r_0 = 3/4 $$, $$a_1 = 1 $$, $$a_2 = 1000 $$ and $$a_\varGamma = a_{\varGamma _1} = 0 $$, with a right-hand side $$f = -\,4$$ and $$f_\varGamma = 0$$. The boundary conditions were symmetry boundaries at $$x = 0$$ and $$y = 0$$ and Dirichlet boundary conditions corresponding to the exact solution at $$x = 1$$ and $$y = 1$$. This example is outlined in Figs. 5 and 6. We give the elevation of the approximate solution in Fig. 7, on the last mesh in a sequence. The corresponding convergence of the $$ L_2$$-norm and the energy-norm is given in Fig. 8.

### Example 2: Flow in the fracture

**Fig. 9:**
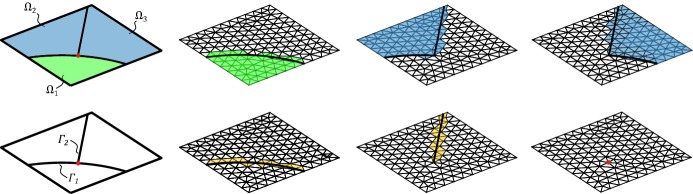
Active meshes with two embedded fractures, Example 2

**Fig. 10:**
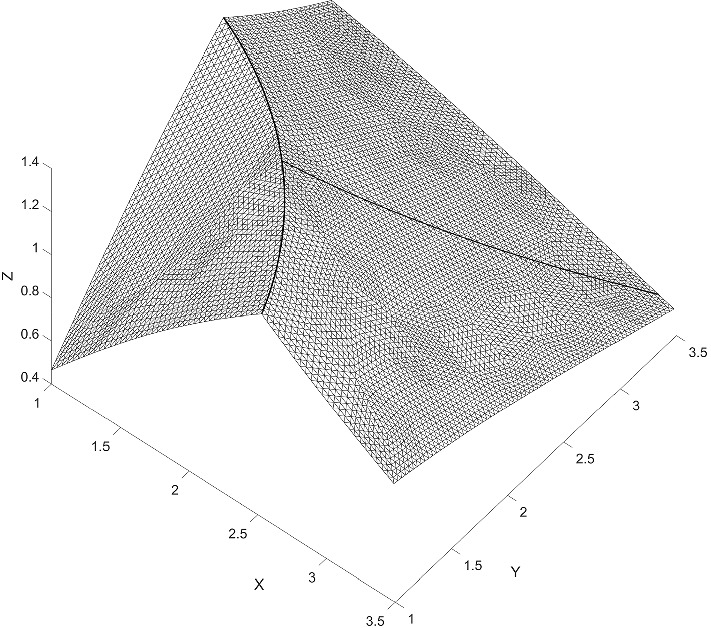
Elevation of the approximate solution with two embedded fractures, Example 2

**Fig. 11:**
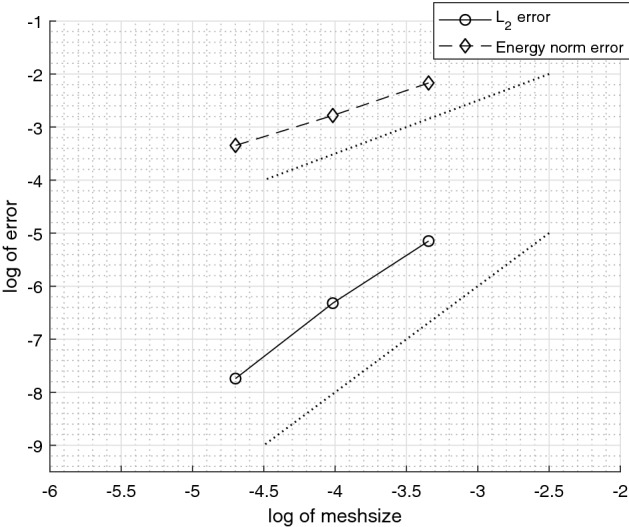
$$ L_2$$-norm and energy-norm convergence using natural logarithm with two embedded fractures, Example 2. Dotted lines signify optimal convergence. Inclination 1:1 for energy-norm and 2:1 for $$ L_2$$-norm

We considered a two-dimensional example on the domain $\varOmega = \left( 1, e^{5/4} \right) \times \left( 1, e^{5/4} \right)$, from Burman et al. (2017). We solved the example with an additional fracture added, see Fig. 9. The exact solution is given by

$$\begin{aligned} \begin{aligned} u_1&= \frac{\log \left( r\right) }{5} \left( 4 + e\right) \quad \text {for } \quad 1< r< e, \\ u_2&= \frac{4 - 4e}{5} \left( \log \left( r\right) - \frac{5}{4}\right) + 1 \quad \text {for } \quad e< r < e^{5/4}, \end{aligned} \end{aligned}$$

where $$\sqrt{x^2 + y^2} := r = e$$. We chose $$a_1 = a_2 = a_\varGamma = 1 $$ and the right hand side to $$f = f_\varGamma = 0$$. For the added crack we chose $$a_{\varGamma _1} = 0 $$. The Dirichlet boundary conditions corresponding to the exact solution at $$x,y = 1$$ and $$x,y = e^{5/4} $$. In Fig. 10, we give the elevation of the approximate solution. The corresponding $$ L_2$$-norm convergence and the energy-norm is given in Fig. 11.

**Fig. 12:**
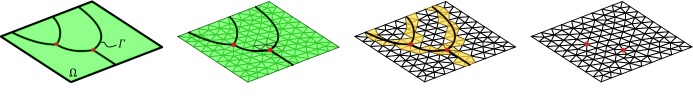
Active meshes with two bifurcating points, Example 3

**Fig. 13:**
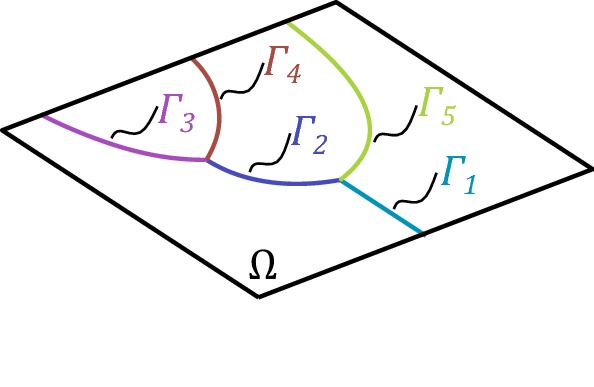
Embedded fractures with assigned $\varGamma$, Example 3

**Fig. 14:**
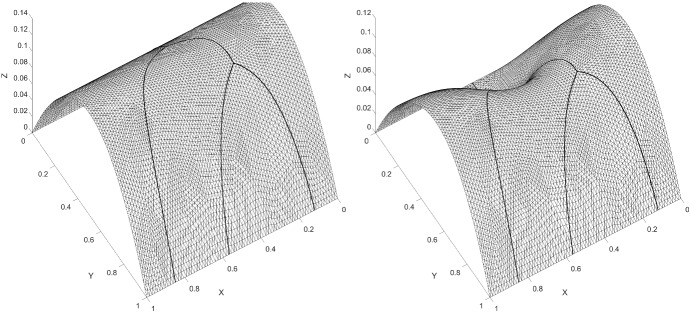
Elevation of the approximate solution using two bifurcating points, Example 3. Assigned value to the left figure: $a_{\varGamma _1} = a_{\varGamma _2} = a_{\varGamma _3} = a_{\varGamma _4} = a_{\varGamma _5} = 0$, and assigned values to the right figure: $a_{\varGamma _1} = 100$ and $a_{\varGamma _2} = a_{\varGamma _3} = a_{\varGamma _4} = a_{\varGamma _5} = 0$

**Fig. 15:**
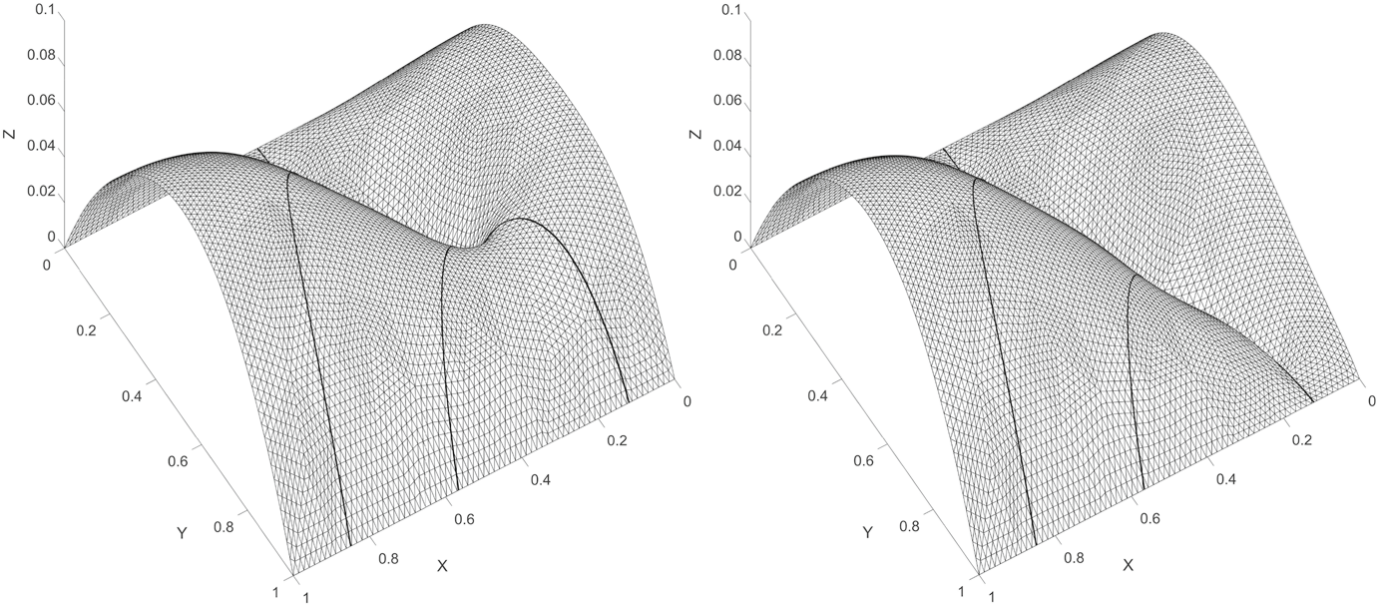
Elevation of the approximate solution using two bifurcating points, Example 3. Assigned value to the left figure: $a_{\varGamma _1} = a_{\varGamma _2} = 100$ and $a_{\varGamma _3} = a_{\varGamma _4} = a_{\varGamma _5} = 0$, and assigned values to the right figure: $a_{\varGamma _1} = a_{\varGamma _2} = a_{\varGamma _3} = 100$ and $a_{\varGamma _4} = a_{\varGamma _5} = 0$

### Example 3: Flow in bifurcating fractures

**Fig. 16:**
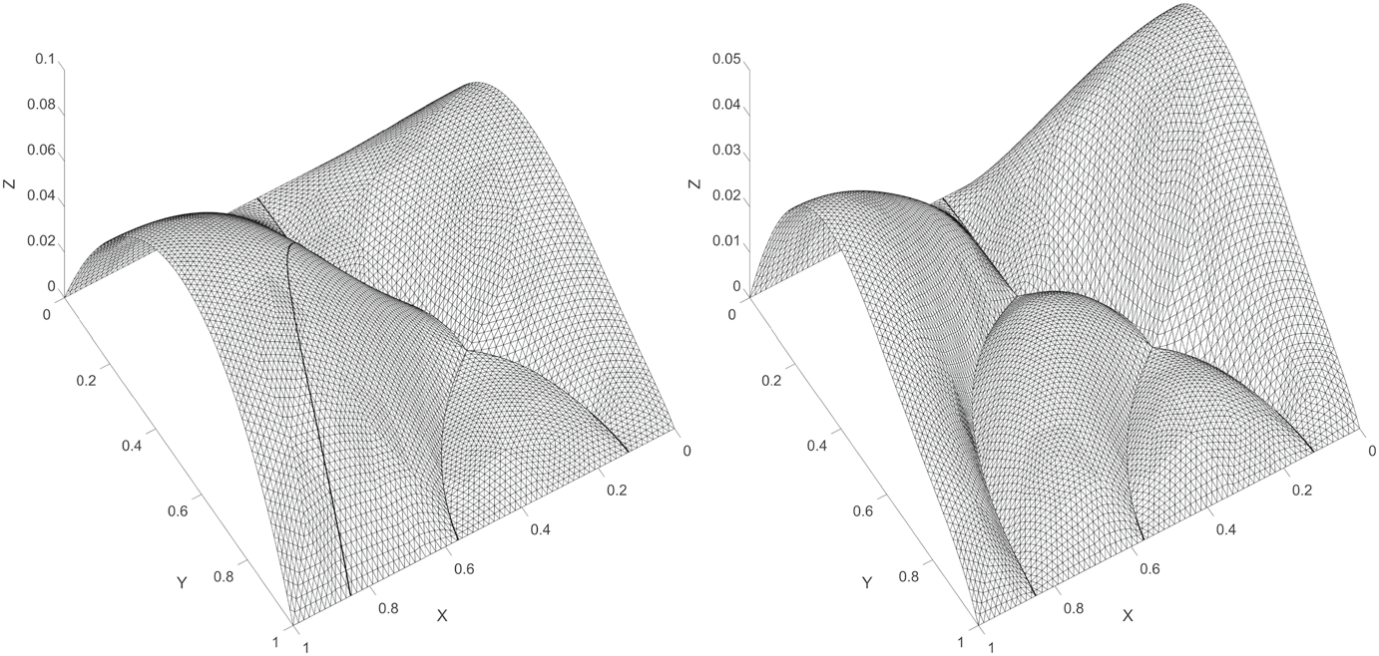
Elevation of the approximate solution using two bifurcating points, Example 3. Assigned value to the left figure: $a_{\varGamma _1} = a_{\varGamma _2} = a_{\varGamma _3} = a_{\varGamma _4} = 100$ and $a_{\varGamma _5} = 0$, and assigned values to the right figure: $a_{\varGamma _1} = a_{\varGamma _2} = a_{\varGamma _3} = a_{\varGamma _4} = a_{\varGamma _5} = 100$

We consider an example with two bifurcating points. The fractures are modeled using higher order curves. In Fig. 12 we show the fractures and construction of individual elements. On the domain $$\varOmega = (0, 1) \times (0, 1)$$, we chose $$ a_1 = a_2 = 1$$, $$ f_\varOmega = 1 $$ and $$f_{\varGamma } = 0 $$. We impose the Dirichlet boundary conditions $$ u = 0 $$ at $$x,y = 0 $$ and $$ u = 0 $$ at $$x,y = 1 $$. For the diffusion coefficient, we denote $$ a_{\varGamma _i} $$ for each fracture where $$ a_{\varGamma _i} \in \{0, 100\}$$ and assign an individual value for each $$ \varGamma _i $$, see Fig. 13. We report six different solutions by allowing different fractures to be active. The first result have been obtained with $$ a_{\varGamma _i} = 0$$, thus no flow in the fractures is allowed, see Fig. 14. Further, each solution has one additional fracture activated by changing the diffusion coefficent $$ a_{\varGamma _i} = 100$$, see Figs. 14, 15 and 16. The last solution is presented with flow in all fractures.

## Concluding remarks

We proposed a discontinuous finite element method using a one–field approach to modelling Darcy flow in a cracked medium. The pressure in the crack was modelled as an average of pressure on either side of the crack which, unlike our previous work (Burman et al. 2017), allows for pressure jumps across the crack. In particular, the case of bifurcating fractures was considered. Optimal order error estimates were proven and backed up by numerical experiments. Extension to other flow models in the crack have been considered in Burman et al. (2018).
